# The ‘critical trochanter angle’ does not show superiority over the CCD angle in predicting varus stem alignment in cementless short-stem total hip arthroplasty

**DOI:** 10.1007/s00402-022-04340-5

**Published:** 2022-01-12

**Authors:** Matthias Luger, Sandra Feldler, Lorenz Pisecky, Jakob Allerstorfer, Tobias Gotterbarm, Antonio Klasan

**Affiliations:** 1 Department for Orthopedics and Traumatology, Kepler University Hospital GmbH, Krankenhausstrasse 9, 4020 Linz, Austria; 2 Johannes Kepler University Linz, Altenberger Strasse 69, 4040 Linz, Austria

**Keywords:** Critical trochanter angle, Stem alignment, Short stem, Total hip arthroplasty, THA, Fitmore, Anterolateral approach

## Abstract

**Purpose:**

Varus positioning of the femoral stem can affect the hip offset (HO). The critical trochanter angle (CTA) was introduced in 2019 as a novel geometric angle, to predict varus stem alignment in cementless straight stem THA. The aim of this study was to evaluate the applicability of the CTA as a predictor for stem alignment in THA with a cementless neck resecting short stem.

**Patients and methods:**

In this retrospective study, 106 patients (index surgery 2014–2019) with unilateral THA and a morphologically healthy contralateral hip as a reference (Kellgren–Lawrence ≤ 1) were included. A cementless short stem with meta-diaphyseal fixation and press-fit cup was implanted in all cases. Stem alignment, CCD angle, CTA and offset reconstruction were measured on preoperative and 3 months postoperative AP radiographs of the pelvis.

**Results:**

Preoperative lower CTA and lower CCD angle were positively correlated (*r* = 0.472; *p* < 0.001). Higher varus stem alignment is correlated with lower CTA (*r* = − 0.384; *p* < 0.001) and lower CCD angle (*r* = − 0.474; *p* < 0.001). A CTA of 23.1° or lower showed a sensitivity of 59.1% and a specificity of 87.1% (AUC: 0.733) and a CCD angle of 132.75° or lower a sensitivity of 68.2% and a specificity of 80.6% (AUC: 0.77) for a varus stem alignment > 3°.

**Conclusion:**

The CTA is also applicable in cementless THA with a neck resecting short stem to evaluate risk of intraoperative varus stem positioning. The CCD angle shows higher sensitivity with marginally lower specificity. Therefore, the CTA is not superior in predicting varus stem alignment in short-stem THA.

**Level of evidence:**

IV.

## Introduction

Correct reconstruction of femoro-acetabular offset and leg length are clinically important factors in total hip arthroplasty (THA) [1]. Conventional straight stems show excellent long-term outcomes [2], but have the disadvantage of limited ability to restore the femoral offset (FO) due to their straight stem design [3]. Restoration of the native FO increases postoperative range of motion, abductor muscle function and decreases polyethylene wear [3–5]. Varus malalignment of the femoral stem in the coronal plane may affect offset or leg length restoration and can hamper optimum load transfer between the implant and natural bone [6]

Haversath et al. [7] have introduced a novel geometric angle named ‘critical trochanter angle’ (CTA) as a predictor for stem alignment. A low CCD angle and a long neck and a trochanter overhand enhance the risk for intraoperative varus stem positioning [7, 8]. The CTA measures the extent of the trochanter overhang in relation to the femoral shaft axis [7]. A CTA lesser or equal to 22.75° showed a sensitivity of 90% and a specificity of 80% for a varus stem position of 2° or greater [7].

The CTA was described in cementless THA with a cementless collarless straight stem design with a narrow shoulder [7]. In recent years, cementless short stems have been increasingly used [1, 9, 10]. Short stems show advantages in the reconstruction of femoro-acetabular offset [1, 11], are superior in preservation of proximal bone stock [12], and facilitate minimally invasive surgery [9, 13]. A short curved stem can initially be inserted in a more varus position following a c-shaped path [9]. However, the final position of a short stem depends on the stem design, fixation, and the level of the neck osteotomy. In case of a neck resecting short stem [14], the aim of the final implantation is oriented in line with the diaphysis [9].

As the insertion of a neck resecting short stem is initially in a more varus position, the risk for final varus malalignment exists. Therefore, this study was conducted to evaluate, if the CTA is also applicable as a predictor for stem alignment in THA with a cementless neck resecting short stem.

## Methods

### Patients

This retrospective radiological comparative study includes patients of a consecutive series of THAs with the same cementless curved short stem (Fitmore® stem, ZimmerBiomet, Warsaw, IN, USA) and bi-hemispherical press-fit acetabular cup (Allofit®/-S, ZimmerBiomet) performed via a minimally invasive supine anterolateral approach. Fitmore® hip stem is a titanium alloy stem (Ti Al6V4) that has a Porolock Ti-VPS coating in the proximal part to enhance bone ingrowth and is available in four different neck angle options (127°, 129°, 137°, 140°) and 14 different sizes (size 1–14) for each offset option [9]. A cementless titanium press-fit cup with or without screws (Allofit®/-S, ZimmerBiomet, Warsaw, IN, USA) was used in all patients.

A consecutive series of 1052 hips in 982 patients with index surgery between 2014 and 2019 were screened for inclusion and the medical records until 90 days postoperative were evaluated. The preoperative X-rays of the pelvis (both hips in comparison, anterior–posterior view, standing upright) were screened for unilateral THA. Diagnoses for inclusion were primary osteoarthritis, avascular necrosis of the femoral head or mild dysplasia of the hip (Crowe I) [15]. Exclusion criteria were defined as bilateral hip disease (Kellgren–Lawrence > grade 1) [16], a history of prior hip surgery, previous trauma, postoperative complication, reoperation, or revision for any reason as well as missing preoperative or postoperative radiographs. In total, 106 patients met the inclusion criteria.

Radiographic measurements were performed on preoperative and 3 months postoperative low centered anteroposterior (AP) radiographs of the pelvis in both groups. Preoperative age at operation, gender, body mass index (BMI), and laterality were recorded.

The study was approved by the institutional review board (EK-No.: 1239/2019). Due to the retrospective study design with evaluation of pre-existing medical records, informed consent was not required. All procedures performed in studies involving human participants were in accordance with the ethical standards of the institutional and/or national research committee and with the 1964 Helsinki declaration and its later amendments or comparable ethical standards.

### Surgical technique and treatment protocol

Surgical procedures were carried out at the author’s institution by surgeons with different levels of experience including 11 consultants and 7 residents. All consultants perform more than 50 and all senior consultants more than 100 arthroplasties per year. Resident surgeries were done under the guidance of a consultant. In all cases, a minimally invasive anterolateral Watson–Jones approach in supine position on a standard operating table under laminar airflow was performed. Extremity preparation was performed with threefold antiseptic scrub with alcohol disinfectant. Draping with a sterile adhesive surgical iodine film was used. The skin incision was centered over the greater trochanter. An incision at the border between the tensor fasciae latae and the tractus iliotibialis was performed. Then the Watson–Jones interval between tensor fasciae latae und gluteus medius was bluntly dissected. A capsulectomy was performed in every case. Fluoroscopy was routinely used with the definitive cup and trial stem in situ. The standardized peri- and postoperative protocol was identical in all cases, including single-shot antibiotics (Cefuroxime 1.5 g i.v. directly preoperative), weight-bearing as tolerated from the first postoperative day on, indomethacin 75 mg daily for the prevention of heterotopic ossification on day 1–4 postoperatively and 40 mg low-molecular weight heparin or Rivaroxaban 10 mg for 28 days postoperatively as venous thromboembolic event prophylaxis.

### Radiographic evaluation

**Fig. 1:**
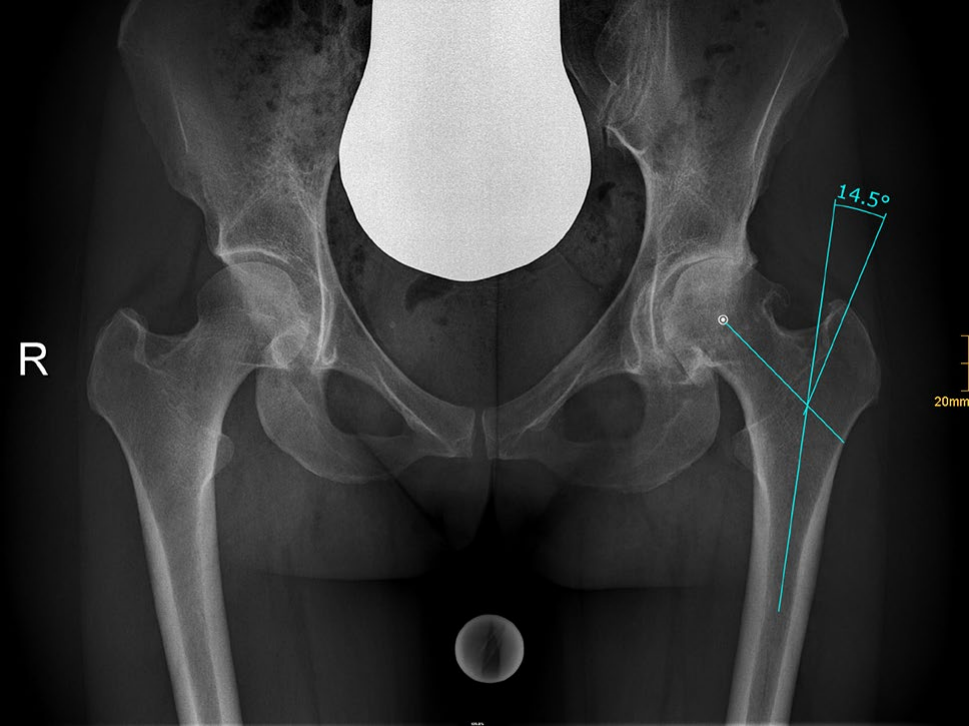
Schematic measurement of the critical trochanter angle (CTA)

**Fig. 2:**
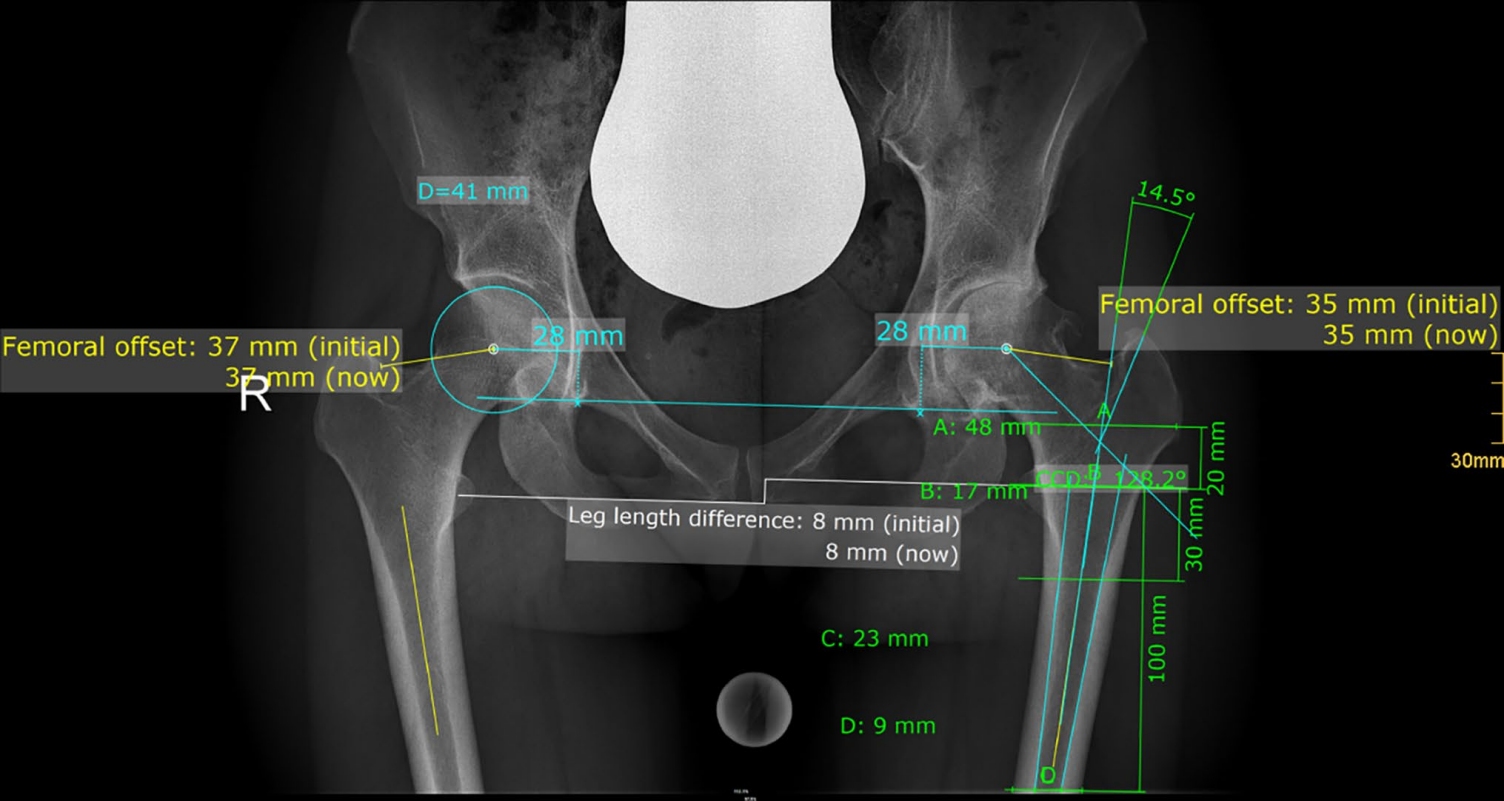
Preoperative measurements: HO, FO, AO, LLD (all bilaterally), CTA, CCD angle, Canal Flare Index, Cortical Index, canal to calcar ratio (all unilaterally)

**Fig. 3:**
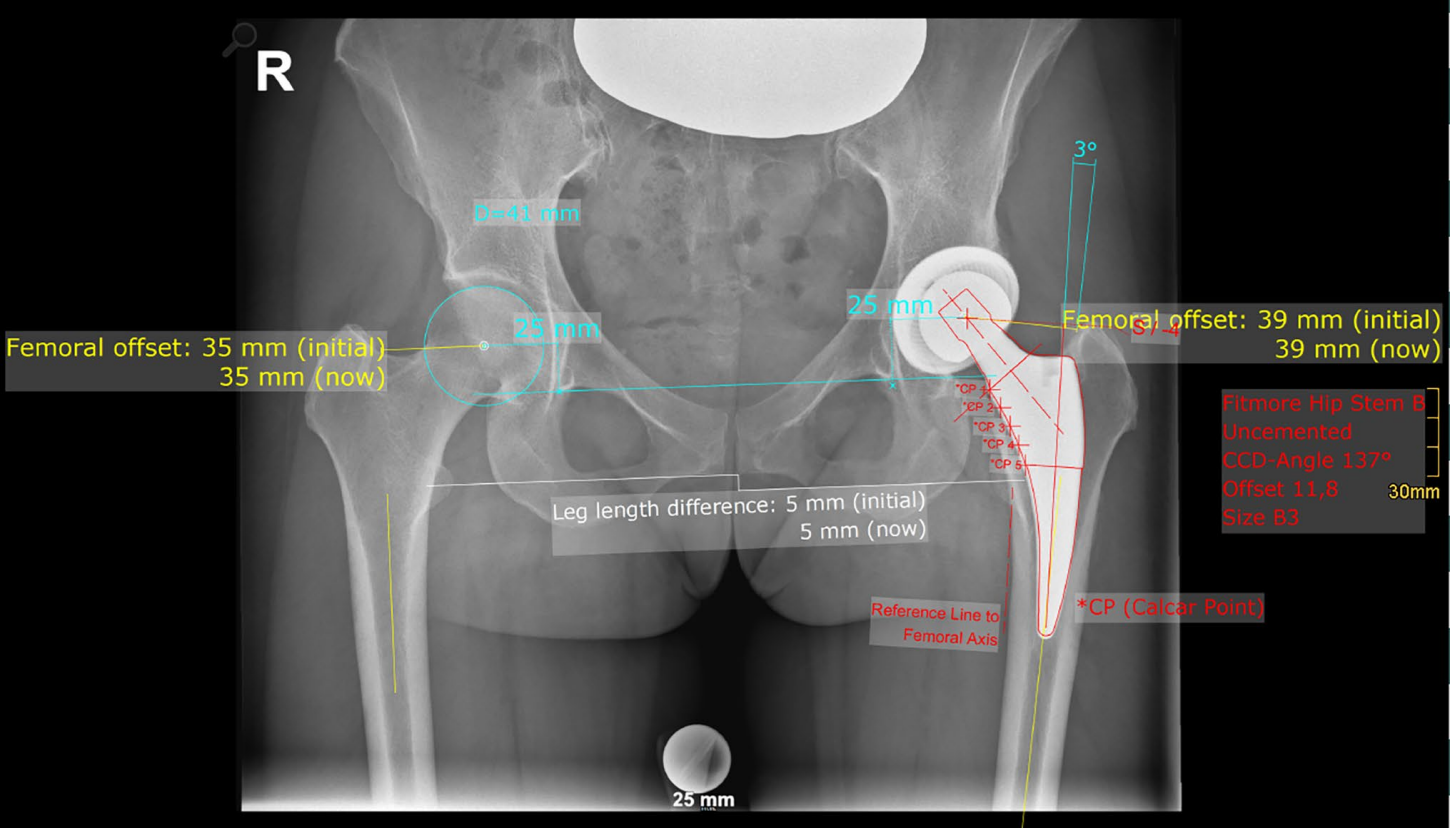
Postoperative measurements: HO, FO, AO, LLD (all bilaterally), stem alignment (unilaterally)

Radiographic measurement was performed on preoperative and 3 months postoperative digital low-centered AP radiographs of the pelvis [17]. Measurements were conducted independently by two reviewers (M.L., J.A.), who were not involved in the index surgery. Radiographs were taken with the patient in standing position and with both legs in 15° internal rotation with the central beam directed on the symphysis pubis [18]. To achieve an accurate measurement of the hip anatomy a double coordinate system was applied on both the preoperative and the postoperative images [1, 19]. Radiographic analysis was performed using MediCAD® Software V5.1 (Hectec GmbH, Germany). The hip center of rotation (COR) was defined using a circle tool determining the diameter of the femoral head and its center [20]. The femoral offset (FO) was determined as the perpendicular distance between the COR and the proximal femoral shaft axis (FSA) [17, 20]. Acetabular offset (AO) was measured as the perpendicular distance between the COR and line T, with T being the perpendicular line on the transteardrop line (TT) through the ipsilateral teardrop figure [17]. Hip offset (HO) was calculated as the sum of FO and AO [17]. The vertical position of the COR was measured as the perpendicular distance to line TT [21]. Radiographic leg length discrepancy (LLD) was measured as the perpendicular distance between line TT and the middle of the lesser trochanter (LT) [18]. Centrum–collum–diaphyseal (CCD) angle was determined according to M. E. Müller on the affected hip [22]. Definition of the stem axis of the implanted cementless stem was previously described for cementless straight stem [23] and for Fitmore® hip stem [24]. For enabling exact measurement of the stem axis of the implanted Fitmore® stem, a digital template of the stem size was put over the implanted stem on the postoperative X-ray. The templating software displayed the correct stem axis of the implanted Fitmore® hip stem, Fig. 3. The critical trochanter angle (CTA) was measured as described by Haversath et al. [7]. The angle crest is defined as the intersection of the femoral shaft axis and the femoral neck axis. A leg between the angle crest and the trochanter vertex is built, and the CTA is measured between this leg and the femoral shaft axis, Fig. 1. To characterize the anatomical shape of the proximal femur and the thickness of cortical bone, the canal to calcar isthmus ratio and the cortical index (CI) according to Dorr et al. [25] were determined. A high CI indicates a thick cortical bone [25]. Additionally, the canal flare according to Noble et al. [26] was determined. The stem alignment was measured as the difference in degrees between the anatomic femoral shaft axis and the vertical stem axis [27]. On preoperative X-rays FO, AO, HO and LLD were measured bilaterally, while CCD angle, CI, Canal Flare Index and canal to calcar ratio were measured unilaterally on the affected hip. Complete preoperative measurements are also shown in Fig. 2.

On postoperative X-rays FO, AO, HO and LLD were measured bilaterally, while stem alignment was measured unilaterally on the operated hip. Complete postoperative measurements are also shown in Fig. 3.

Intra- and interobserver reliabilities were calculated. Intra-class correlation coefficients (ICC) were used with a two-way random effects model for absolute agreement. Repeated measurements for intraobserver reliability were performed at day 1 and day 14 in a blinded fashion.

### Statistical analysis

**Fig. 4:**
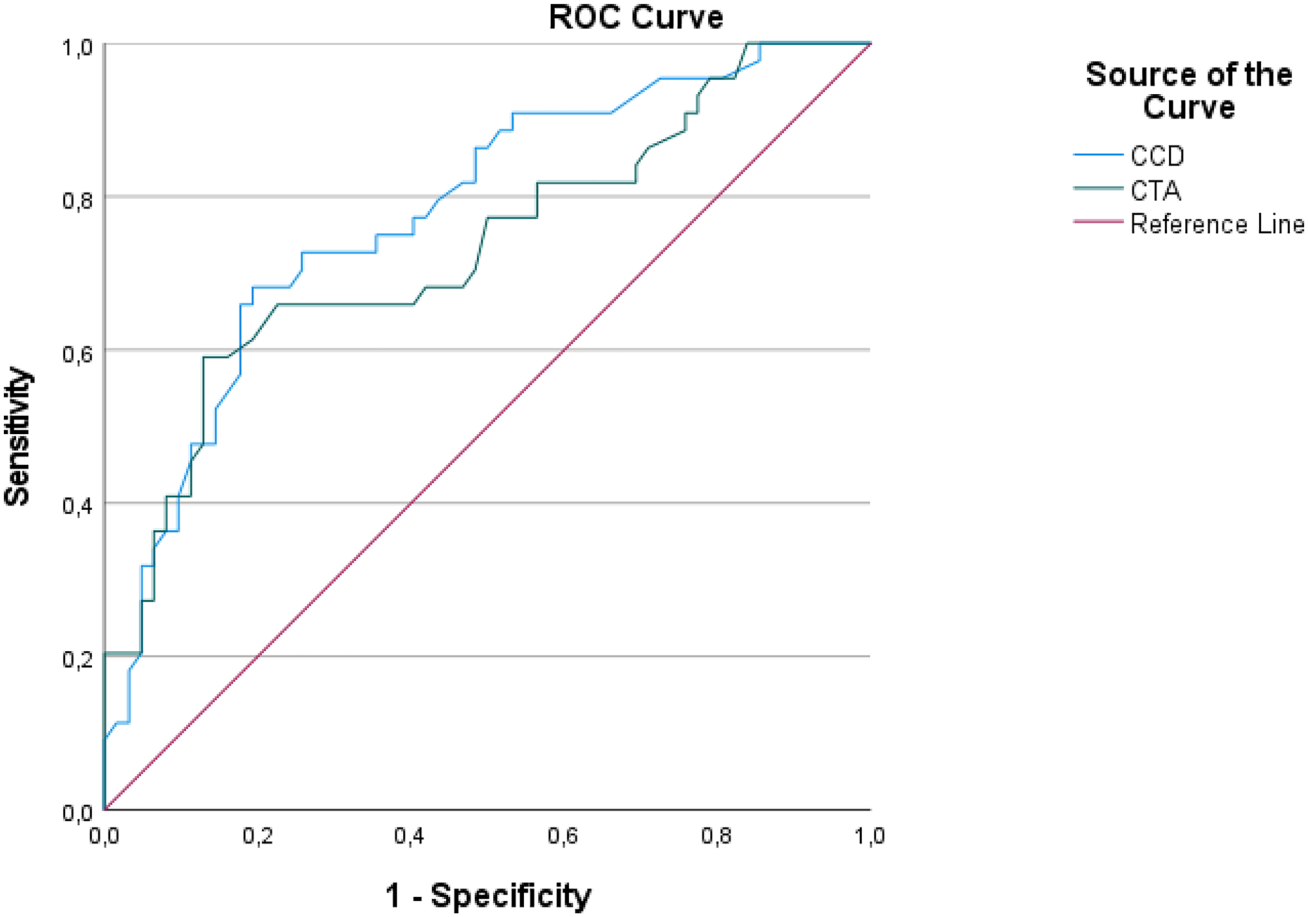
ROC curve for CCD angle and CTA for varus stem alignment > 3°

Descriptive statistical analysis was conducted for age, gender, body mass index (BMI) and laterality, as well as measurements for FO, AO, HO, LLD, Canal Flare Index, CI, canal to calcar ratio, stem alignment, CCD angle and CTA. Power analysis was not performed due to the observed statistical significance for the primary endpoint [28]. An ANOVA was conducted for testing differences in CCD-Angle, CTA and stem alignment. Pearson’s coefficient was used for the correlation of CCD angle and CTA with preoperative FO and HO, Canal Flare Index, CI and canal to calcar ratio. Additionally, a Pearson’s coefficient was used for the correlation of CTA, CCD angle, and stem alignment with postoperative difference in HO and FO to the contralateral side and leg length difference. Preoperative and postoperative differences in HO, FO, and AO were always calculated in relation to the contralateral healthy hip. A receiver operating characteristics (ROC) curve was calculated to obtain an analysis for sensitivity and specificity of CTA und CCD cutoff values that result in varus stem alignment greater than 3°, with the reported area under the curve (AUC) (Fig. 4). The Youden Index was used to define the optimal cut‐point [29]. Statistical analysis was performed with SPSS version 27 (IBM SPSS statistics, Chicago, IL, USA). A p value < 0.05 was considered as statistically significant.

## Results

The intraobserver interclass correlation coefficient between the two sets of measurements was 0.992% (95% confidence interval 0.987–0.994, *P* < 0.001).

The patient demographics are shown in Table 1. Average CCD angle and CTA were significantly lower (*p* < 0.001) in patients with varus stem alignment, Table 2. FO and HO differences were also significantly higher (*p* = 0.006; *p* = 0.024) in patients with postoperative varus stem alignment.

**Table 1:** Patient demographics

Number of patients	106
Gender (male:female)	41:65
Side (left:right)	49:57
Age at operation (years)	57.11 ± 11.17
BMI (kg/m^2^)	27.61 ± 4.95

**Table 2:** Preoperative and postoperative measurements

FO preoperative (mm)	39.46 ± 6.67	
FO difference preoperative (mm)	1.93 ± 4.12	
AO preoperative (mm)	34.58 ± 4.43	
AO difference preoperative (mm)	0.82 ± 2.46	
HO preoperative (mm)	74.04 ± 8.48	
HO difference preoperative (mm)	1.11 ± 4.08	
Canal flare index	3.91 ± 0.633	
Cortical index	0.6 ± 0.05	
Calcar to canal ratio	0.57 ± 0.07	
Stem alignment (°)	3.1 ± 4.65	** < 0.001**
0°–1°	18 (17.0%)	
> 1–5°	44 (41.5%)	
> 5°	44 (41.5%)	
CTA (°)	20.07 ± 6.88	** < 0.001**
0°–1°	24.41 ± 7.93	
> 1–5°	21.4 ± 5.89	
> 5°	16.97 ± 6.05	
CCD (°)	131.41 ± 6.53	** < 0.001**
0°–1°	136.77 ± 5.91	
> 1–5°	132.56 ± 5.84	
> 5°	131.41 ± 6.53	
HO difference postoperative (mm)	2.92 5.39	**0.024**
0°–1°	0.44 5.2	
> 1–5°	2.48 4.54	
> 5°	4.39 5.89	
FO difference postoperative (mm)	6.74 6.61	**0.006**
0°–1°	5.06 6.06	
> 1–5°	5.02 5.53	
> 5°	9.14 7.17	
AO difference postoperative (mm)	− 3.81 4.15	**0.028**
0°–1°	− 4.61 5.09	
> 1–5°	− 2.55 3.53	
> 5°	− 4.75 4.053	

Bold letters for significant values

A significant correlation was found for CTA and CCD angle, preoperative FO and HO, Table 3. A lower CTA was correlated with lower CCD angle (*r* = 0.472; *p* < 0.001). A negative correlation was found for CTA and preoperative FO (*r* = − 0.522, *p* < 0.001) and HO (*r* = − 0.421, *p* < 0.001), meaning a lower CTA correlated with higher preoperative FO and HO. A significant correlation for CCD angle and CTA (*r* = 0.472; *p* < 0.001), preoperative FO (*r* = − 0.569; *p* < 0.001), HO (*r* = − 0.418; *p* < 0.001), Canal Flare Index (*r* = − 0.252; *p* = 0.009), Cortical Index (*r* = − 0.418; *p* < 0.001) and canal to calcar ratio (*r* = 0.219; *p* = 0.024) was found.

**Table 3:** Correlation of preoperative measurements with CTA and CCD angle

CTA	CCD angle	FO preoperative	HO preoperative	Canal flare index	Cortical index	Canal to calcar ratio
Pearson’s *r*	**0.472**	− **0.522**	− **0.421**	− 0.080	− 0.147	0.011
*P* value	** < 0.001**	** < 0.001**	** < 0.001**	0.414	0.131	0.911

CCD	CTA	FO preoperative	HO preoperative	Canal flare index	Cortical index	Canal to Calcar ratio
Pearson’s *r*	**0.472**	− **0.569**	− **0.418**	− **0.252**	− **0.418**	**0.219**
*P* value	** < 0.001**	** < 0.001**	** < 0.001**	**0.009**	** < 0.001**	**0.024**

Bold letters for significant values

Table 4 shows the correlation for postoperative measurements. A lower CTA is significantly correlated with a lower CCD angle (*r* = 0.472; *p* < 0.001) and with higher varus stem alignment (*r* = − 0.384; *p* < 0.001). A lower CCD angle is correlated with higher varus stem alignment (*r* = − 0.474; *p* < 0.001). Higher varus stem alignment is significantly correlated with lower CTA (*r* = − 0.384; *p* < 0.001) and CCD angle (*r* = − 0.474; *p* < 0.001). A significant correlation is also for increased postoperative FO (*r* = 0.724; *p* < 0.001) and HO (*r* = 0.357; *p* < 0.001).

**Table 4:** Correlation of postoperative measurements and BMI with CTA, CCD angle and stem alignment

CTA	CCD	Stem alignment	HO difference post-OP	FO difference post-OP	Leg length difference	BMI
Pearson’s *r*	**0.472**	− **0.384**	< 0.000	− 0.031	− 0.024	0.103
*P* value	** < 0.001**	** < 0.001**	0.999	0.751	0.807	0.294

CCD	CTA	Stem alignment	HO difference post-OP	FO difference post-OP	Leg length difference	BMI
Pearson’s *r*	**0.472**	− **0.474**	− 0.056	0.022	0.028	0.032
*P* value	** < 0.001**	** < 0.001**	0.567	0.823	0.777	0.746

Stem alignment	CTA	CCD	HO difference post-OP	FO difference post-OP	Leg length difference	BMI
Pearson’s *r*	− **0.384**	− **0.474**	**0.357**	**0.724**	− 0.077	− 0.175
*P* value	** < 0.001**	** < 0.001**	** < 0.001**	** < 0.001**	0.435	0.072

Bold letters for significant values

The ROC analysis showed an AUC of 0.77 for CCD angle and 0.733 for CTA for a varus stem alignment > 3°. The Youden Index was highest for a CTA of 23,1° with a sensitivity of 59.1% and a specificity of 87.1% for a varus stem alignment > 3. The Youden Index was highest for a CCD angle of 132.75° with a sensitivity of 68.2% and a specificity of 80.6% for a varus stem alignment > 3.

## Discussion

A lower CTA and CCD angle is significantly correlated with higher varus stem alignment in cementless THA with a neck resecting short curved stem. Additionally, higher varus stem alignment leads to significantly higher increase in FO and HO compared to a contralateral healthy hip as reference. Also, a positive correlation is found for higher varus stem alignment and postoperative FO and HO difference.

Haversath et al. [7] initially described the CTA as a predictor for risk of varus stem alignment in cementless THA with a cementless collarless straight stem design with a narrow shoulder for direct anterior approach (DAA) and lateral Hardinge approach. A statistically significant correlation was found for CTA and CCD angle. A coxa vara deformity was significantly correlated with lower CTA [7]. Murphy et al. [8] also described a higher varus stem alignment in cementless straight stem THA in patients with coxa vara deformity. The results in the present study show comparable findings. A significant correlation between lower CTA and lower CCD angle was also found.

A lower CTA is negatively correlated with higher varus stem alignment in straight stem THA [7]. A lower CCD angle is also negatively correlated with higher varus stem alignment [7, 8]. These findings are also applicable in short-stem THA. Our results show similar statistically significant correlations between CCD angle, CTA and stem alignment. Therefore, a lower CCD angle and CTA are also possible risk factors for varus stem alignment in cementless short-stem THA with a neck resecting short stem. Although the stem used in this study has a C-shaped curvature, designed to minimize varus malalignment, this study demonstrates that it nevertheless does occur, with the risks being similar to those for the straight cementless stems.

The cut-off of 22.75° or less for the CTA showed a sensitivity of 90° and specificity of 80° for varus stem alignment of 2° or greater in straight stem THA [7]. We detected a cut-off for the CTA for a varus stem alignment > 3° of 23.1° with a sensitivity of 59.1% and a specificity of 87.1%. A cut-off of 132.75° for the CCD angle showed a higher a sensitivity of 68.2%, but a lower specificity of 80.6% for a varus stem alignment > 3. In contrast to Haversath et al. [7] we defined the varus malalignment of greater than 3° of varus because in case of a short curved stem we believe that a more varus positioning is more common because of the c-shaped calcar-guided insertion of rasps. We found similar cut-off values for CTA with 23.1° compared to 22.5° [7]. However, sensitivity in the presented study is significantly lower with 59.1% compared to 90° [7]. The specificity is comparable with 87.1% compared to 80° [7]. A cut-off 132.75° for the CCD angle showed a higher sensitivity with minimally lower specificity. Therefore, we conclude that the CCD angle is as reliable for predicting varus stem alignment as the CTA in short-stem THA with a curved short stem.

A higher varus stem alignment is associated with a higher increase of HO and FO compared to a contralateral healthy hip as reference. A correlation between higher varus stem alignment and postoperative leg length difference could not be found. Testing for postoperative HO and FO difference resulted in a statistically significant difference in postoperative HO and FO difference with higher varus stem alignment with > 1° or > 5° of varus placement of the femoral stem. A postoperative difference in HO ≥ 5 mm compared to a contralateral hip is associated with poorer delta gain in Harris Hip Score (HHS). These significantly lower delta gains increase with every 2.5 mm increase above 5 mm. The results in the presented study show an increase in postoperative HO of 2.48 mm (± 4.54 mm) and 4.39 mm (± 5.89 mm) in patients with varus positioning between > 1° and 5° and > 5°. Therefore, the clinical impact of higher varus stem positioning might be without high clinical relevance. These results are similar compared to previous studies, reporting a moderate increase in HO and FO with a varus alignment of Fitmore® stem ≥ 3° [30]. Additionally, a mild varus stem alignment of a cementless short stem does not lead to an increased risk of periprosthetic fracture [31]. As the impact of mild varus stem alignment leads to minor increases in HO and might not lead an increased risk in perioperative complications it might be rather radiographically unsatisfying rather than clinically important. However, our results do not include patient-orientated outcome measurements (PROMs) and, therefore, a final verdict cannot be given with our presented data.

Several limitations of the study have to be addressed. First, we tried to minimize a potential selection bias with very strict inclusion criteria. Only patients with a single implant design and approach were included in this study. The results might not be applicable to other stem designs, although our results are similar to those with a straight stem. A homogenous study cohort was created by excluding patients with a bilateral hip disease (Kellgren–Lawrence > grade 1). Furthermore, we aimed to increase reliability of the measurements and results by restricting inclusion based on preoperative diagnosis. We excluded all forms of secondary osteoarthritis of the hip and development dysplasia of the hip Crowe grade II to IV. Prior surgery before THA was also excluded. However, mild hip dysplasia (lateral center–edge angle 20–25°), coxa profunda, and morphologic alterations related to cam- or pincer-type impingement were included, because these changes might be subtle and cannot be reliably identified in the present study cohort with end-stage disease. Therefore, we conclude that the findings in the present study are applicable for primary osteoarthritis and care must be taken when applying our findings on secondary osteoarthritis or high grades of development dysplasia of the hip. Second, we address the fact of taking measurements on plain radiographs. FO is underestimated by approximately 13% on plain radiographs [20]. However, our measurements are easily reproducible, applicable in daily routine and less invasive regarding radiation exposure. Furthermore, we postulate variances in inter- and intraobserver reliability in measuring clinical leg length difference. Additionally, this study lacks of missing clinical outcome scores or PROMs. However, aim of this study was to find out, if the CTA is also applicable in cementless short-stem THA, which could be achieved by the presented data. We are fully aware that further research is necessary to any clinical relevance of varus placement of cementless short stems.

## Conclusion

The CTA is also applicable in cementless THA with a neck resecting short stem to evaluate risk of intraoperative varus stem positioning. The CCD angle shows higher sensitivity with marginally lower specificity. Therefore, the CTA is not superior in predicting varus stem alignment in short-stem THA.

## Data Availability

Data and materials are available on request.
